# Anthropometric and physiological profiles of highly trained sailors in various positions and levels

**DOI:** 10.1038/s41598-024-62160-6

**Published:** 2024-05-17

**Authors:** Dandan Pan, Kaiyang Sun, Xiuxia Liu

**Affiliations:** 1 Shanghai Elite Sport Training Administrative Center, Shanghai, 202162 China; 2 Shanghai Research Institute of Sports Science, Shanghai, 200030 China; 3 Xiamen University, Amoy, 361005 China

**Keywords:** Anthropometric profiles, Physiological profiles, Sailors, Helmsman, Crew, Physiology, Health care

## Abstract

This study aimed to analyze anthropometric and physiological profiles of highly trained sailors and the differences between sailors regarding various training levels. Forty-two sailors (22 male, 22.4 ± 3.8 years; 20 females, 21.3 ± 3.6 years) were divided into helmsmen and crew groups, and the high- and low-level were distinguished. Sailors completed height, sitting height, legs length, weight, BMI, VO2max, 30 s all-out sprint, isometric mid-thigh pull (IMTP), countermovement jump, bench pull, core endurance tests. The results showed the crew had higher height, sitting height, weight, VO2max and lower trunk flexor endurance test times compared to the helmsmen (*p* < 0.05). The helmsmen had higher relative peak power/force in the 30 s all-out sprint and IMTP tests compared to the crew, whereas the crew had better absolute strength in bench pull, with significant differences between female sailors (*p* < 0.05). The high-level sailors showed more sailing experience than low-level sailors (*p* < 0.05). In conclusion, highly trained crew tend to be taller and heavier, while helmsmen have better trunk flexor endurance. For female sailors, helmsmen have better lower-body power and strength and crew have better upper-body strength. Sailing experience is a reliable variable to distinguish sailors’ levels. The specific anthropometric and physiological profiles of sailors in various positions can assist sailing coaches in athlete selection and intervention training.

## Introduction

Sailing is a skills-oriented endurance sport involving the boat moving through the water driven by the wind. Sailors are not only required to give full play to their own abilities, but also to simultaneously focused on the environmental conditions, opponents’ positions and maneuvers during the regatta^1,2^. Successful performance is influenced by a variety of factors such as anthropometry, physiology, technique and tactics^3,4^. Olympic sailing consists of classifications such as single-handed (only one sailor manipulates the boat) and double-handed (two sailors jointly manipulate the boat) dinghy^5^, according to the position of the sailors on the boat can also be divided into helmsmen and crew. The role tasks that sailors need to complete vary according to the sailing class and position on the boat^6^. Both the 470 and 49er classes are double-handed dinghy events in which the helmsman is primarily responsible for operating the mainsail and the steering gear (the basis for changing direction) to keep the boat moving in the right direction during sailing^7,8^. As the wind increases, the helmsman “hiking out” from the side of the boat assists the crew in counteracting the boat’s tilting to maintain balance. The crew mainly works the jib sail and spinnaker (high intensity loaded rope-pulls) and adjusts the inclination of the hull to the left or the right sides, while repeatedly perform rapid body position changes to keep the boat speed and overall performance^8,9^. However, the sailor of a single-handed dinghy such as laser are required to perform the tasks of helmsman and crew alone, including: steer the boat; sheet the mainsail according to the wind; move fore and aft to adjust the trim; hike to keep the boat flat; move fore and aft to adjust the trim; watch the wind, water, and waves, and make sure the boat is taking the right course, and so on^7,10^.

Anthropometric characteristics are defined as properties are responsible for the dynamics of growth and development, which help optimize competitive performance and monitor training regimens, and play an important role in determining the success of an athlete^11^. Previous studies have been shown that different positions and different classes of sailors have different anthropometric characteristics^8,12^. The anthropometric indicators can help athletes choose the suitable sailing class and position, which plays a vital role in effective control the boat and sailing performance. Furthermore, sailors constantly adjust their body positions and maneuvers to keep the boat steady and thus increase its speed during sailing, which requires sailors to have well-developed physical components (strength, endurance, balance) and body function (anaerobic and aerobic)^13^. In recent years, in order to improve the competition and enjoyment of sailing competitions, skiffs and hydro-foiling sailing classes have been introduced and new regatta formats have been developed, which means that sailors’ physical and physiological demands increased dramatically^14^. In order to be successful in a race, the sailors must have the optimal anthropometric requirements as well as good physical fitness characteristics.

A clear understanding of favorable profiles (anthropometry and physiology) plays an important role in enhancing training effectiveness and optimizing performance, and may also help in talent identification and the development of personalized training programs^15^. Considering that this information is scarce in various positions and levels of highly trained sailors, the purposes of this study are to identify anthropometric and physiological profiles of highly trained sailors and to analyze possible differences in anthropometric and physiological parameters, age, sailing experience of helmsmen and crew regarding various training levels.

## Methods

### Participants

Forty-two highly trained sailors (22 male, 20 female) were selected and divided into helmsmen group (n = 30, including ILCA 6 [n = 7], ILCA 7 [n = 8], 470 helmsmen [n = 9], 49er and 49erFX helmsmen [n = 6]) and crew group (n = 12, including 470 crew [n = 6], 49er and 49erFX crew [n = 6]) based on their position. Sailors were also classified by training level: high-level group (Male: helmsmen [n = 8], crew [n = 3]; female: helmsmen [n = 6], crew [n = 2]) and low-level group (Male: helmsmen [n = 7], crew [n = 4]; female: helmsmen [n = 9], crew [n = 3]). The high-level group consisted of international or national level athletes, and the low-level group consisted of athletes of other grade levels. All the sailors have more than 5 years sailing training experience, and training time is higher than 25 h per week. Participants were instructed to avoid any strenuous exercise during the 24 h preceding each testing session. Participants were informed of all experimental procedures and written informed consent was completed before participation. The study protocols were approved by the Capital University of Physical Education and Sports Ethics Committee and according to the ethical principles of the World Medical Association Declaration of Helsinki.

### Procedures

#### Anthropometric measures

Anthropometric measurements were completed by specialized personnel of Shanghai Research Institute of Sports Science in strict accordance with the detailed rules of “Sports Measurement and Evaluation”. Height, sitting height and leg length were measured with an appropriate stadiometer to the nearest 0.1 cm. Body weight was obtained with a digital scale (TANITA, HD-366, Japan) without shoes and wearing minimal clothes, to the nearest 0.1 kg. The body mass index (BMI) was calculated as weight divided by the square of the height^15^.

#### Physiological tests

Physiological tests included: 1) Aerobic and anaerobic capacity: maximal oxygen uptake, 30 s all-out sprint; 2) Strength and power: isometric mid-thigh pull, countermovement jump, bench pull; 3) Core endurance: flexor endurance, extensor endurance.

#### Maximal oxygen uptake

An incremental test was performed on a rowing ergometer (Concept II, USA) to assess aerobic capacity. During the test, several respiratory parameters were analyzed by COSMED metabolic systems (COSMED, K5, Italy). The experimental protocol was as follows: an incremental progressive exercise test starting from 120/90 W(male/female) for 2 min and increased by 30/20 W(male/female) every 2 min until the participant felt exhausted^16^. The test was stopped when the participant had three of the following four states conditions: 1) Respiratory exchange ratio greater or equal to 1.10; 2) Heart rate (HR) in excess of 90% of age predicted HRmax (HRmax = 220 − age); 3) An identification of a plateau (< 150 ml/min increase) in VO_2_max. 4) The participant could not maintain exercise with the required load^17^. The VO_2_max and relative values of VO_2_max were recorded after test.

## 30s all-out sprint

The anaerobic capacity test was performed on an air-braked cycle ergometer (Wattbike Pro, Nottingham, UK). The participant carried out an incremental load cycling (2.0 W/kg for 7 min, then 2.5 W/kg, 3.0 W/kg, 3.5 W/kg for 3 min) and 3 short sprints (3 s maximal sprints with 20 s of easy pedaling between) for warm-up. After completing the warm-up, the participants took a rest for 1–3 min before starting official test. The sprint test implemented a “5-s rolling start” before the 30 s maximal sprint. The tester continued to give verbal encouragement and feedback on elapsed time. The computer attached to the cycle ergometer was used to record peak power, mean power relative peak power, relative mean power during the sprint test^18^.

## Isometric mid-thigh pull (IMTP)

IMTP test was performed on squat rack with a force plate (Kistler 5695BQ2, Kistler Instrumente, Switzerland) to examine lower-body strength. The bar height was adjusted up or down to allow the participant to obtain the optimal knee (130–150°) and hip (140–160°) angles. The sailor was instructed to assume proper body position: feet roughly centered under the bar approximately hip width apart, knees underneath and in front of the bar, and thighs in contact with the bar, upright torso, shoulder girdle retracted and depressed, hands holding the bar (lifting strap can be used). Before the test, the participant performed 3–5 s submaximal attempts^19^. Then participant performed 2–3 trails, each trial lasting 5 s. A third trial was only used if the different of ≥ 250 N was observed between the first two trials. The participant was instructed as “fast and hard pull” as possible, and received loud, verbal encouragement performed. Peak force and relative peak force were recorded^20^.

## Countermovement jump (CMJ)

CMJ test was performed on a force plate (Kistler 5695BQ2, Kistler Instrumente, Switzerland) to examine lower-body power. Each participant completed 3 attempts with 1 min of rest between trials. Participants started from the standing position with their hands on the hips to prevent any influence of arm movements, flexed the hips and knees after hearing a countdown of “3, 2, 1, jump”, immediately followed by extension of these joints and jump as high as possible. Jump height, peak force and peak power were recorded (normalized to body mass)^21^.

## Bench pull

Bench pull test was performed with a standard Olympic barbell and bench pull rack to evaluate upper-body strength. After normal warm up, familiarization was conducted through a self-determined, exercise-specific warm up consisting of 3–4 sets of the bench pull exercise using progressively heavier loads. The participant was instructed to lie prone on the bench and grasp an Olympic bar with a pronated grip, elbows fully extended, head, trunk and legs in contact with the bench. A repetition was considered valid when the barbell touched the bottom of the bench^22^.

## Core endurance

Core endurance test is performed on a bench, including trunk flexor and extensor endurance. Trunk extensor endurance was performed using the Biering-Sorensen test^23^. The participant was instructed to lie prone on a bench with all body parts above his/her anterior superior iliac spines hanging off the edge of bench, crossing his/her arms in front of chest and lifting his/her upper body up until his/her trunk was horizontal to the ground, holding lower extremities onto the bench with the help of a partner^24^. Combined with the maximal hiking maneuver characteristics of sailors, trunk flexor endurance was conducted using the movement in the opposite direction to extensor endurance test^25^. As we can see from supplementary information (flexor endurance test). The participant was instructed to lie supine on a bench with all body parts above his/her anterior superior iliac spines hanging off the edge of bench, maintaining the hip angles at 160–180°, with a “warning line” set above the participant’s chest, which the participant was not allowed to touch, holding lower extremities onto the bench with the help of a partner. Time was recorded until participant could no longer control his/her posture for a maximum of 300 s.

### Statistical analysis

All data were presented with mean ± standard deviation and analyzed with the SPSS Statistics V26.0 software (IBM Corporation, Armonk, NY). Normal distribution of data was tested using Shapiro–Wilk test, and homogeneity was performed with Levene’s test. The differences in variables between helmsman and crew (the normally distributed data) were compared with Independent Samples T Test, the data which did not distribute normally were compared with Mann Withney U test. Effect sizes (d) were estimated by calculating the 95% confidence intervals for Cohen’s d and interpreted as follows: trivial (≤ 0.20), small (0.20 to < 0.60), moderate (0.60 to < 1.20), large (1.20 to < 2.00), very large (2.00 to < 4.00), and extremely large (≥ 4.00)^26^.

## Results

The demographic information and anthropometrical parameters of all sailors were presented in Table 1. The results showed significant difference between sailors in various position, the crew had higher values in terms of height, sitting height and weight than helmsmen, and male crew had longer legs length and female crew had higher BMI (*p* < 0.05, d = 1.18–2.51).

**Table 1:** Demographic information and anthropometric parameters in the groups of helmsmen and crew.

Variables	Sex	Samples		*p*	d	ILCA (n = 15)	470		49er	
		Helmsmen (n = 30)	Crew (n = 12)				Helmsmen (n = 8)	Crew (n = 6)	Helmsmen (n = 7)	Crew (n = 6)
Age (years)	M	22.0 ± 3.7	23.1 ± 4.1	0.239	0.30	21.0 ± 3.3	21.8 ± 3.6	23.7 ± 3.9	24.0 ± 3.2	22.8 ± 3.7
	F	21.4 ± 3.6	21.0 ± 3.7	0.722	0.11	21.1 ± 4.0	21.5 ± 3.2	20.7 ± 3.8	22.0 ± 2.2	21.5 ± 2.5
Sailing experience (years)	M	10.7 ± 5.3	7.6 ± 3.7	0.236	0.63	8.1 ± 5.3	12.5 ± 4.4	8.0 ± 3.6	13.3 ± 3.3	7.3 ± 3.3
	F	10.0 ± 5.4	9.2 ± 6.0	0.709	0.14	9.1 ± 5.6	11.0 ± 4.6	9.3 ± 6.1	11.0 ± 4.2	9.0 ± 4.0
Height (kg)	M	178.6 ± 5.3	189.1 ± 4.4	0.001**	2.10	182.1 ± 3.7	173.9 ± 3.9	187.1 ± 2.2	177.1 ± 3.5	190.6 ± 4.5
	F	167.8 ± 6.6	174.9 ± 1.5	0.030*	1.21	171.9 ± 4.1	162.4 ± 6.2	175.3 ± 1.3	164.2 ± 3.5	174.3 ± 1.3
Sitting height (cm)	M	96.6 ± 2.2	101.7 ± 1.7	0.001**	2.51	97.5 ± 1.7	94.9 ± 1.5	101.4 ± 1.7	96.5 ± 2.3	101.9 ± 1.4
	F	91.2 ± 3.1	95.0 ± 1.2	0.018*	1.35	92.4 ± 2.4	89.3 ± 3.8	94.7 ± 1.2	90.7 ± 1.3	95.5 ± 0.6
Legs length (cm)	M	91.2 ± 3.7	95.6 ± 3.9	0.018*	1.18	93.8 ± 2.2	87.8 ± 3.3	94.4 ± 2.5	90.0 ± 1.4	96.5 ± 4.0
	F	84.3 ± 4.4	88.5 ± 2.1	0.059	1.04	87.5 ± 2.6	80.2 ± 1.6	89.0 ± 2.1	81.4 ± 3.3	87.7 ± 1.2
Weight (kg)	M	72.6 ± 7.2	82.3 ± 4.5	0.001**	1.51	78.6 ± 3.8	64.9 ± 1.9	78.4 ± 2.2	69.7 ± 4.8	85.3 ± 2.4
	F	60.7 ± 5.7	70.8 ± 2.3	0.001**	1.96	64.5 ± 3.1	56.4 ± 4.7	70.2 ± 2.4	56.4 ± 4.0	71.6 ± 0.6
BMI (kg/m^2^)	M	22.7 ± 1.8	23.1 ± 1.6	0.692	0.19	23.7 ± 1.5	21.5 ± 1.0	22.4 ± 1.0	22.3 ± 1.8	23.6 ± 1.6
	F	21.6 ± 1.8	23.1 ± 1.0	0.050*	1.20	21.9 ± 1.7	21.3 ± 0.6	22.9 ± 1.0	20.9 ± 1.7	23.6 ± 0.6

*M* male; *F* female; *p*: significant difference between the helmsmen and crew, * p < 0.05, ** p < 0.01; d: effect size.

The results of the physiological tests were presented in Table 2. From the aerobic test, the crew showed significantly higher values in VO_2_max than the helmsman (*p* < 0.01, d = 1.44–1.77), but there was no difference in relative values of VO_2_max. In terms of 30 s all-out sprint and IMTP tests, we found the helmsmen presented a higher relative peak power and relative peak force compared to the crew, while the crew performed better in bench pull absolute strength, and there were significant differences between female sailors (*p* < 0.05, d = 1.20–1.85). Moreover, the helmsman had better flexor endurance than the crew (*p* < 0.05, d = 0.96–1.09).

**Table 2:** The results of physiological tests in the groups of helmsmen and crew.

Variables		Male		*p*	d	Female		*p*	d
		Helmsmen (n = 15)	Crew (n = 7)			Helmsmen (n = 15)	Crew (n = 5)		
VO_2_max	VO_2_max (L/min)	4.26 ± 0.48	4.90 ± 0.34	0.005**	1.44	3.14 ± 0.24	3.58 ± 0.28	0.003**	1.77
	VO_2_max (ml/min/kg)	58.87 ± 5.05	59.29 ± 4.31	0.852	0.09	52.40 ± 3.79	50.20 ± 2.95	0.255	0.61
30 s all-out sprint	Peak power (W)	1035.20 ± 134.01	1115.57 ± 93.18	0.169	0.65	715.20 ± 88.39	766.40 ± 54.17	0.242	0.62
	PP_W_ (W/kg)	14.11 ± 1.34	13.50 ± 1.10	0.148	0.48	11.92 ± 0.93	10.85 ± 0.70	0.032*	1.20
	Mean power (W)	629.67 ± 71.73	682.43 ± 66.34	0.116	0.75	427.60 ± 53.37	455.00 ± 36.69	0.304	0.55
	MP_W_(W/kg)	9.68 ± 2.58	8.24 ± 0.58	0.162	0.66	7.22 ± 0.80	6.44 ± 0.42	0.054	1.06
IMTP	Peak force (N)	2989.27 ± 538.46	3209.71 ± 328.82	0.333	0.45	2012.73 ± 222.95	2096.60 ± 205.48	0.486	0.38
	PF_W_ (N/kg)	41.10 ± 5.33	38.93 ± 2.65	0.113	0.46	33.47 ± 2.71	29.70 ± 3.30	0.020*	1.32
CMJ	Jump height (cm)	41.57 ± 4.56	40.91 ± 4.51	0.680	0.15	31.10 ± 3.52	30.34 ± 3.68	0.684	0.21
	PF_W_ (N/kg)	245.78 ± 34.99	245.46 ± 30.67	0.755	0.01	227.32 ± 19.39	219.86 ± 17.06	0.631	0.39
	PP_W_ (W/kg)	53.37 ± 6.01	53.74 ± 4.15	0.984	0.07	47.20 ± 4.85	45.94 ± 4.53	0.614	0.26
Bench pull	Absolute strength (kg)	82.00 ± 9.36	86.43 ± 5.56	0.091	0.53	55.17 ± 4.17	62.70 ± 3.73	0.006**	1.85
	Relative strength (kg/kg)	1.13 ± 0.09	1.05 ± 0.07	0.052	0.95	0.91 ± 0.09	0.89 ± 0.05	0.531	0.33
Core endurance	Flexor endurance (s)	146.87 ± 40.95	109.71 ± 32.36	0.040*	0.96	167.47 ± 60.91	107.20 ± 27.96	0.019*	1.09
	Extensor endurance (s)	192.40 ± 45.83	154.57 ± 42.91	0.219	0.84	188.00 ± 36.93	162.00 ± 28.70	0.055	0.74

*PP*_*W*_ relative peak power; *MP*_*W*_ relative mean power; *IMTP* isometric mid-thigh pull; *PF*_*W*_ relative peak force; *p*: significant difference between the helmsmen and crew, * p < 0.05, ** p < 0.01; d: effect size.

**Figure 1:**
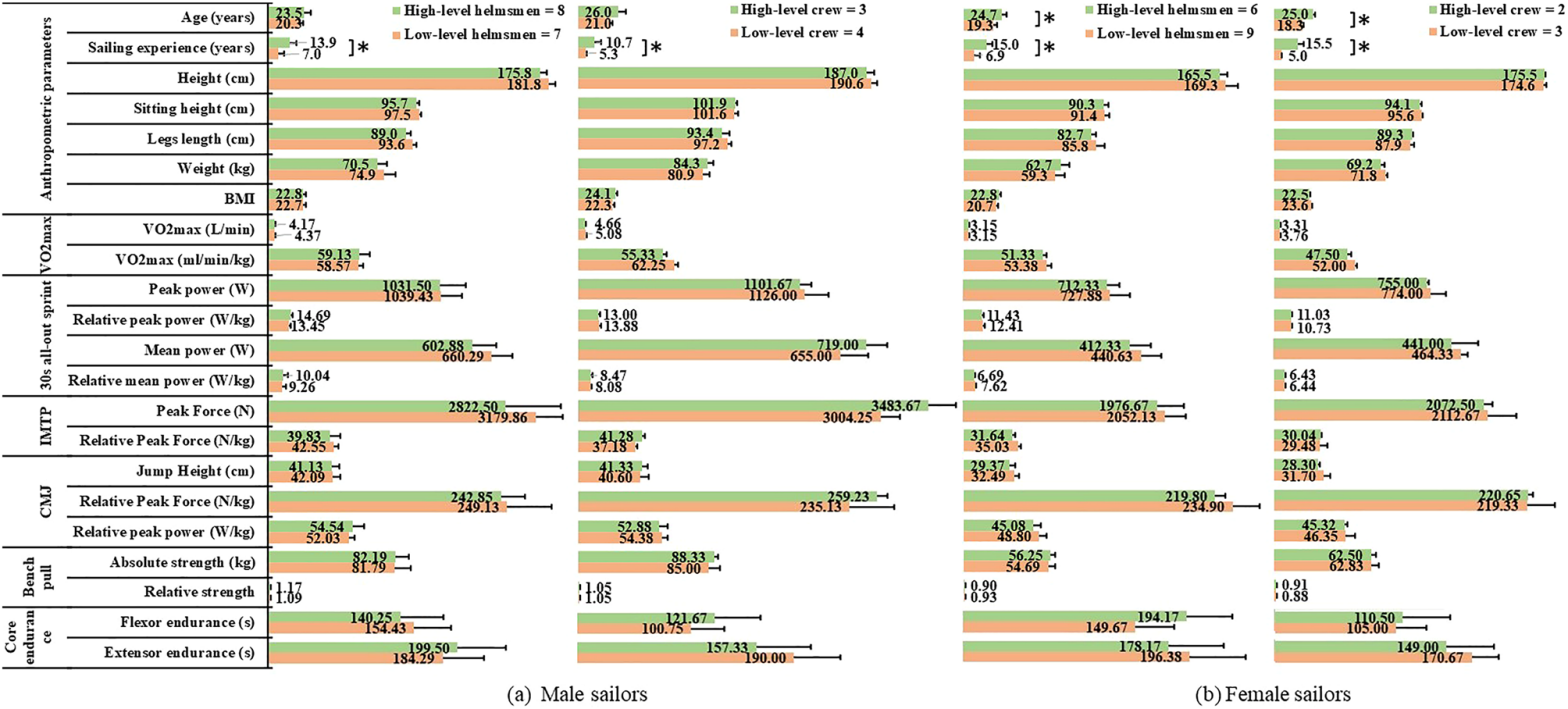
The results of age, sailing experience, anthropometric and physiological parameters in the groups of high-level and low-level sailors. *: significant difference between high and low levels group of sailors (*p* < 0.05).

Differences in age, sailing experience, anthropometric and physiological parameters between high- and low- levels are presented in Fig. 1. Regarding sailing experience, the high-level sailors showed significantly higher values than low-level sailors, and high-level female sailors are older compared to low-level female sailors (*p* < 0.05). No significant differences were found in rest of the parameters between high-level and Low-level sailors.

## Discussion

Anthropometric and physiological measurements may possibly be used to detect potentially successful athletes in specific sports. The current study attempts to describe the anthropometric and physiological profiles of highly trained sailors and to evaluate possible differences in age, sailing experience, anthropometric and physiological parameters of helmsman and crew regarding various training levels, with the intentions of assisting sailing coaches to identify or screen talents and conduct intervention training.

This study found that the crew had higher height, sitting height and weight than helmsmen, and male crew also had longer legs length. Considering that previous studies have proved that the sailors from each sailing class need different anthropometric characteristics, it is improper to look for a single optimal profile^4,27^. We discuss the anthropometric parameters of sailors of different classes separately. According to Skrypchenko^8^, Prlunda^28^ research on Olympic sailors, it can be found that the height and weight of sailors were significant differences among sailors in different classes, while there were within a specific range among sailors in the same position. The obtained values can serve as modal characteristics in sailors and make the selection of the athletes easier. Fletcher^29^ suggested that the height and weight of elite sailors were as follows, Laser: 178–188 cm and 78–82 kg, Laser Radial: 166–176 cm and 66–68 kg, 470 male helmsmen/crew: 172–176 cm and 58 kg/180 cm and 70—72 kg, 470 female helmsmen/crew: 160–176 cm and 58 kg/172–177 cm and 70 kg, 49er helmsmen/crew: 174–180 cm and 68–73 kg/180–188 cm, 75–80 kg. Prlenda^28^ examined the anthropometric characteristics of sailors in 8 sailing classes from the 2008 to 2016 Olympics, and found that the height and weight of the first 15 best placed sailors were around the average of a specific range, and the average was gradually increasing.

There is some disagreement about the effect of height and weight on sailing performance. A view is expressed that the performance during the race has a relatively high sensitivity to the weight of the sailor, and heavier weight can enable sailors to better cope with strong winds and other restrictions on sailing^30^. Tan^25^ showed that weight was a much stronger determinant of maximal hiking performance than height, probably because the musculoskeletal structure “lever arm” of taller sailors was longer, which imposed a greater load on their muscles and made it difficult for them to fully extended their bodies during hiking. Yet another view is that the sailor’s height and leg length are the key to affecting their performance. Higher height and leg length will allow the sailor to move its center of mass far away from the hull, resulting in a longer lever arm when hiking, thereby generating a greater force moment to counteract the heel of the boat^31^. In general, sailing is a height-dependent and weight-dependent activity, the combination of height and weight has a positive contribution to forming the optimal righting moment during sailing^13^. We need to find a perfect balance between height, weight, and performance based on different classes and positions.

Demographic information and anthropometric parameters of top 10 sailors in Tokyo 2020 Olympic Games are given in Table 3. The data comes from the online platform https://www.olympedia.org/athletes and https://tokyo2020.sailing.org/results-centre. Overall, the helmsmen were shorter (male: 179.2 ± 6.0 cm versus 184.0 ± 5.5 cm, female:167.6 ± 6.9 cm versus 175.8 ± 4.3 cm) and lighter (male: 73.7 ± 8.2 kg versus 77.1 ± 4.8 kg, female: 62.1 ± 5.9 kg versus 69.6 ± 2.0 kg) than crew, while BMI values were similar. Although, the optimal anthropometric requirements differ among these boat classes, the height and weight of elite sailors at the same position in a given class were within a specific range. The results were consistent with our findings. According to Skrypchenko^8^, an analysis applying the BMI classification system recommended by the World Health Organization observed that the average BMI of sailors at the 2016 Olympics was 22.8 ± 1.8 kg/m^2^, similar to many other Olympic-type sports (Olympic athletes in various sports was 22.9 kg/m^2^), and sailors had minimal dispersion of BMI indicators. Appropriate morphological characteristics are favorable factors in determining the success of top sailors in sailing races. It was worth noting that single-handed dinghy sailors (ILCA 6 and ILCA 7) had higher values in terms of height and weight than other helmsmen, closer to the crew. This is because single-handed dinghy sailors need to generate appropriate righting moment by hiking out the side of the boat to maintain the balance during sailing. Especially when sailing in strong wind, higher height and weight can not only help sailors form a greater hiking force, but also reduce physical effort, which directly affects sailing performance^8^. In terms of double-handed dinghy sailing (470, 49er and 49erFX), the work of helmsman and crew are very well defined: the helmsman controls the board and occasionally helps in hiking the boat, and the crew maintain the boat in an upright position by trapezing technique. Crew are attached to a wire on the mast via a metal hook and hang off the edge of the boat, exert a righting force by keeping their bodies further out of the deck to counteract the moment generated by the wind on the sails, thereby facilitating an efficient drive of the boat^32^.

**Table 3:** Demographic information and anthropometric parameters of top 10 sailors in Tokyo 2020 Olympic Games.

Variables	Sex	Samples		ILCA (n = 20)	470		49er	
		Helmsmen (n = 60)	Crew (n = 40)		Helmsmen (n = 20)	Crew (n = 20)	Helmsmen (n = 20)	Crew (n = 20)
Age (years)	M	32.9 ± 5.4	31.9 ± 4.9	32.1 ± 6.4	33.2 ± 5.8	33.6 ± 4.9	33.3 ± 4.8	30.1 ± 4.6
	F	31.0 ± 4.2	29.9 ± 4.1	29.8 ± 3.1	32.3 ± 5.9	31.2 ± 5.5	30.9 ± 3.4	28.6 ± 1.8
Sailing experience (years)	M	24.3 ± 5.5	22.9 ± 4.6	23.8 ± 5.8	25.4 ± 5.6	24.4 ± 4.7	23.6 ± 5.7	21.3 ± 4.3
	F	24.0 ± 4.3	20.6 ± 4.4	23.4 ± 3.1	23.8 ± 5.5	21.1 ± 6.1	24.7 ± 4.4	20.0 ± 2.2
Height (kg)	M	179.2 ± 6.0	184.0 ± 5.5	185.5 ± 3.6	172.6 ± 3.2	182.4 ± 7.1	179.6 ± 2.4	185.6 ± 3.2
	F	167.6 ± 6.9	175.8 ± 4.3	174.6 ± 5.6	161.9 ± 4.1	176.8 ± 4.1	166.2 ± 4.3	174.8 ± 4.7
Weight (kg)	M	73.7 ± 8.2	77.1 ± 4.8	81.6 ± 2.5	63.4 ± 1.8	72.5 ± 1.3	76.2 ± 4.3	81.7 ± 1.6
	F	62.1 ± 5.9	69.6 ± 2.0	68.3 ± 1.7	55.9 ± 3.3	68.5 ± 1.6	62.2 ± 4.2	70.7 ± 1.9
BMI (kg/m^2^)	M	22.9 ± 1.6	22.8 ± 1.5	23.7 ± 1.2	21.3 ± 0.9	21.9 ± 1.6	23.6 ± 1.4	23.7 ± 0.7
	F	22.1 ± 1.3	22.6 ± 1.1	22.5 ± 1.7	21.3 ± 1.3	21.9 ± 0.7	22.5 ± 0.6	23.2 ± 1.2

*M* male; *F* female.

The training response elicited by the long-term training of a given exercise mode is directly related to the physiological elements involved in coping with the specific exercise stress, thus forming the specific physiological profiles. The specific physiological adaptation of training is different between helmsman and crew due to the different position and the role tasks^33^. As we know, helmsman is mainly responsible for steering and making tactical decisions, while crew undertakes more of the physical work and control of the sails. Except for the single-handed dinghy sailors, who are helmsmen, but who have to complete the tasks of helmsman and crew alone to in order to keep the boat sailing properly.

There seems to be a consensus that aerobic capacity is an important requirement for Olympic sailing. As pointed out in the Bojsen-Møller’s study, the physiological demands vary with boat designs and sailor’s position, but sailing is not a weight-bearing activity, so it may be more reasonable to use relative values of VO_2_max to assess sailors’ aerobic capacity^34^. Some authors consider that rowing is the most applicable aerobic activity for sailors because it meets the sailors’ needs for strength, power and aerobic fitness^6,27,29^. Therefore, the rowing ergometer VO_2_max test was performed to monitor the sailors’ aerobic capacity in this study. Our results showed that the crew had a higher VO_2_max values, while there was no difference between the relative values of VO_2_max of the helmsmen and crew groups. According to Serranor^33^, sailors without trapeze keep the boat balance by extending their bodies out to the side of the boat (hiking), which was the most physically demanding in sailing, thus the sailors without trapeze had a higher VO_2_max compared to those that use it. Different from this view, Walker^35^ proposed that the sailors from different classes and positions showed many similarities in skills and performance characteristics, and elite sailors usually had well-developed physical profiles, which may lead to the physiological parameters of elite sailors had many similarities, and the result of this study was consistent with this view. A study on elite sailors reported VO_2_max was 54.65 ± 4.68 ml/kg/min for Laser, 55.76 ± 4.09 ml/kg/min for 470 sailors, with no significant difference between the two classes in terms of aerobic capacity^36^. Another study on elite-level 49er sailors found that the VO_2_max of two groups of national team athletes was 56.3 ± 4.7 ml/kg/min (4.45 ± 0.35 L/min) and 58.5 ± 3.9 ml/kg/min (4.80 ± 0.35 L/min), respectively^9^. Currently, in some studies, the VO_2_max of elite male sailor ranges from 52 to 61 ml/kg/min, while there are fewer studies related to female, with the average VO_2_max of good Danish female sailors being 50.1 ± 1.4 ml/kg/min^36–38^. Based on the above research it can be concluded that our sailors exhibit a superior aerobic capacity.

The present data indicated that the helmsmen exhibited better relative peak power in the anaerobic capacity test, and there was a significant difference between female helmsmen and crew groups. The sailing sport is on the basis of aerobic energy supplying, thought the anaerobic energy supply is few parts of necessary energy in race, it is used as an energy source several times^39^. For instance, when sailors leave the start line or attempt to overtake opponents in strong winds, especially in choppy and gusty conditions, they will use a fully extending hiking movement. Although this movement lasts for a short time, it is repeated many times. Hiking increases the oxygen demand of the exercise muscles, while reduces quadriceps muscle oxygen availability arises from restricted muscle blood flow^13,40^. As a result, there is a mismatch between oxygen supply and demand during hiking, leading to an increase in the attributable of anaerobic capacity. The importance of anaerobic capacity to sailing performance has gradually attracted attention. According to Vangelakoudiet^41^, anaerobic capacity of Laser sailors had an impact on their sailing performance, and the nationally ranked sailors’ mean and maximal anaerobic powers were significantly correlated with their national ranking positions. Our previous research found that the peak and mean power of Chinese elite Laser athletes were 1101.9 ± 174.0 W, 641.7 ± 68.7 W and those of Laser Radial athletes were 639.6 ± 138.9 W, 400.5 ± 54.7 W, respectively^13^. In this study, the peak and mean power of our male helmsmen were slightly lower than the average level of Chinese elite Laser sailors, those of the female helmsmen were higher than the average level of elite Laser Radial sailors.

Furthermore, our study examined the relative lower-body strength of helmsmen was superior to that of the crew, while the absolute upper-body strength of helmsmen was inferior to the crew, and there was a significant difference between female groups. For single-handed dinghy sailors and the helmsmen of 470, the hiking during upwind and reaching sailing is the major physical challenge, and the hiking performance is an important determinant of race result^42^. When sailing in strong winds, the helmsmen use the toe straps to hike over the side and suspend the rest of the body over the water, so as to counter the moment generated by the wind on the sail to tilt the hull and keep the boat as upright as possible, which imposes essentially isometric stress on the quadriceps^41^. Studies have shown that hiking is a dynamic and aerobic movement technique with isometric moments, which is superimposed with jerks approximate the maximum voluntary contraction on the background of isometric contraction^43^. For 49er class, the physical demands placed on the helmsman are minimal when compared to the sailors in other position. In light wind, the helmsman constantly swinging in and out on the wing of the boat via the trapeze, which stress on the quadriceps, like doing a high number of body weight squats. In moderate or strong wind, the helmsman wiring on the trapeze and try to remain as static as possible^29^. Therefore, it seems to be of great importance for the helmsmen to have well-developed lower-body strength. Sailors with a higher maximal strength in the quadriceps use a lower percentage of their maximal strength to maintain hiking postures, which can reduce insufficient the blood and oxygen supply caused by intramuscular pressure, thereby delaying muscle fatigue^44^. However, for the crews, they usually undertake a higher loads of upper limb activities. Raising the kite and trimming the spinnaker are important and hard parts of the crew’s role and take approximately 5–7 s on all-out effort, which require powerful upper-body pull strength and power^29^. Thus, the crew may have superior upper-body strength to helmsmen.

Core endurance refers to the ability to produce sub-maximal muscle actions over extended periods. It plays an important role in controlling spine stability and maintaining an efficient position for trunk, and generating and transmitting force^45^. In this study, the helmsmen performed better in trunk flexor endurance. Research has demonstrated, the hiking postures changes mainly in the sagittal plane from the trunk upright to leaning backwards until the hip is fully extended, with the angle of the helmsmen’s trunk leaning backwards increases, the load on the trunk flexor muscle continues to increase, while the crew are suspended from a wire attached to the mast with the lower back supported by the trapeze harness^42^. This is less pressure on the helmsmen’s core muscles than hiking out. The specific biomechanical requirements and physiological demands to maintain the hiking position may drive the increases trunk flexor endurance of the helmsmen^46^.

The results of comparative analysis of sailors in various levels showed that sailing experience was the key indicator to distinguish the sailors of different levels, and they were positively correlated. According to Serrano^33^, the difference between professional sailors and novices was not due to their “hardware” characteristics, but to their “software” characteristics. The “hardware” referred to the physical and physiological characteristics, while the “software” referred to the technical and tactical skills, knowledge of the rules of the race, familiarity with race field conditions and the ability to analyze situations, stimulus perception and decision-making, and a series of other variables related to the sailing experience. Sailing as a sport conditioned by the environment in which it developed (sea conditions, wind direction and intensity) and the actions of opponents^47^. According to the changes wind and waves and other factors, sailors need to perform the corresponding maneuvers, while according to the opponent’s position and course planning requires the selection of the appropriate tactics. Sailing regatta opponents are facing each other and look for opportunities to interfere with each other. Well-experienced sailors will grab a good position and travel at a faster speed that can avoid boats interfering or covering. They will choose a course that is shorter and faster to the finish line, and will not allow other boats to have access to better course as far as the rules allow, while changing tactics if necessary^39^. High-level sailors spend more time on the water than low-level sailors, with more sailing experience (boat control, technique and tactics, environment and rules understanding), which will enable them to develop the necessary resources to respond to the stimuli during training and competition^48^.

One of the main limitations of our study was the sample size. When we classified the sailors, it would be interesting to have a larger sample of the crew. Another limitation is that we divide sailors into helmsmen and crew by position and analyzed them by the role tasks, but there are other classifications as well.

## Conclusions

Highly trained crew tend to be taller and heavier, while helmsmen have better trunk flexor endurance. For female sailors, helmsmen have better lower-body power and strength and crew have better upper-body strength. Sailing experience is a reliable variable to distinguish sailors’ levels. The specific anthropometric and physiological profiles of sailors in various positions can assist sailing coaches in athlete selection and intervention training.

### Supplementary Information

Supplementary Information.

## Data Availability

All data generated and analyzed during this study are included in this published article.
