# Supplementary figures and images for: Anthropometric and physiological profiles of highly trained sailors in various positions and levels
Source: Sci Rep. 2024 May 17;14:11321. doi: 10.1038/s41598-024-62160-6 (PMC11101428; doi:10.1038/s41598-024-62160-6)

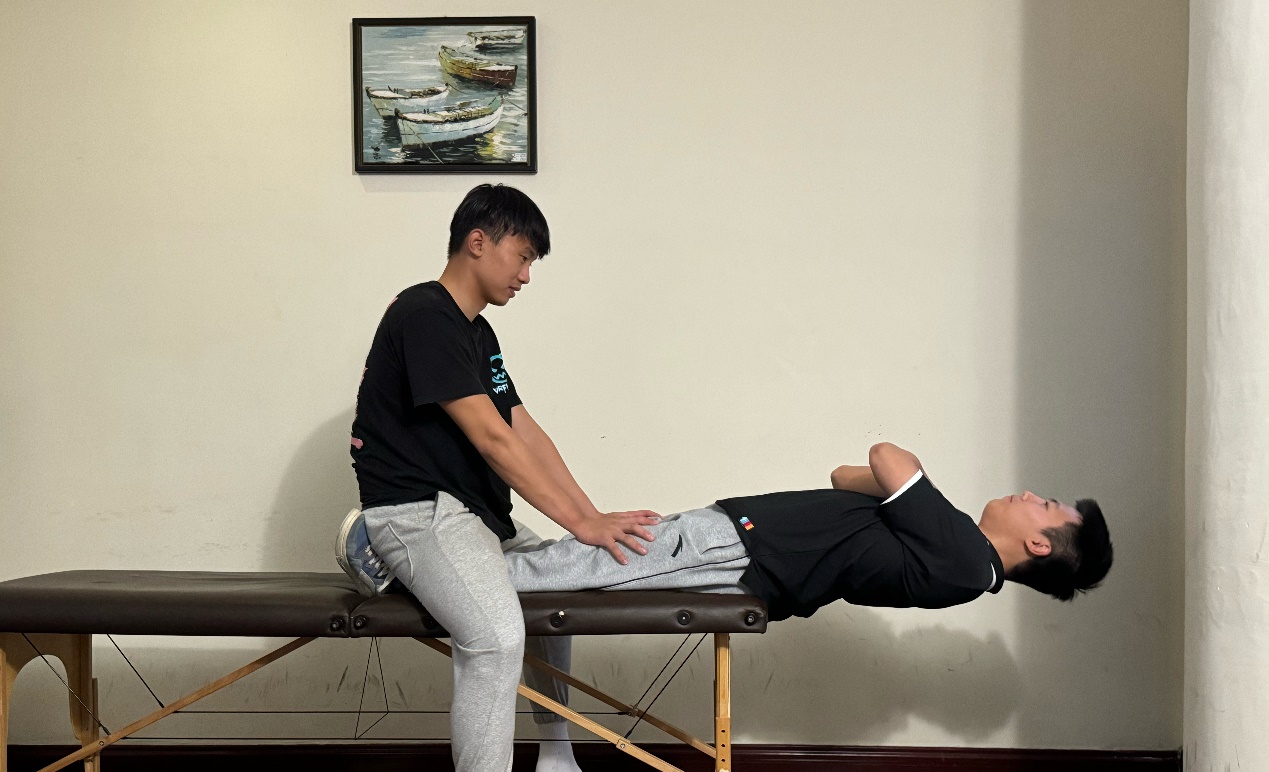
Supplementary figure legend

Figure. Flexor endurance test

Supplement: Supplementary file 1 — Supplementary Information. [file 41598_2024_62160_MOESM1_ESM.docx]
